# Elevated Cardiac Troponin in Acute Stroke without Acute Coronary Syndrome Predicts Long-Term Adverse Cardiovascular Outcomes

**DOI:** 10.1155/2014/621650

**Published:** 2014-11-04

**Authors:** Farhan Raza, Mohamad Alkhouli, Paul Sandhu, Reema Bhatt, Alfred A. Bove

**Affiliations:** ^1^Department of Medicine Cardiology Section, Temple University Hospital, Philadelphia, PA 19140, USA; ^2^Temple University School of Medicine, Philadelphia, PA 19140, USA; ^3^Department of Medicine, Internal Medicine Section, Temple University Hospital, Philadelphia, PA 19140, USA

## Abstract

*Background*. Elevated cardiac troponin in acute stroke in absence of acute coronary syndrome (ACS) has unclear long-term outcomes.* Methods*. Retrospective analysis of 566 patients admitted to Temple University Hospital from 2008 to 2010 for acute stroke was performed. Patients were included if cardiac troponin I was measured and had no evidence of ACS and an echocardiogram was performed. Of 200 patients who met the criteria, baseline characteristics, electrocardiograms, and major adverse cardiovascular events (MACE) were reviewed. Patients were characterized into two groups with normal and elevated troponins. Primary end point was nonfatal myocardial infarction during follow-up period after discharge. The secondary end points were MACE and death from any cause.* Results*. For 200 patients, 17 patients had positive troponins. Baseline characteristics were as follows: age 63.1 ± 13.8, 64% African Americans, 78% with hypertension, and 22% with previous CVA. During mean follow-up of 20.1 months, 7 patients (41.2%) in elevated troponin and 6 (3.3%) patients in normal troponin group had nonfatal myocardial infarction (*P* = 0.0001). MACE (41.2% versus 14.2%, *P* = 0.01) and death from any cause (41.2% versus 14.5%, *P* = 0.017) were significant in the positive troponin group.* Conclusions*. Elevated cardiac troponin in patients with acute stroke and no evidence of ACS is strong predictor of long-term cardiac outcomes.

## 1. Background

The relationship between acute stroke and coronary artery disease is complex, and they are related to each other in multiple ways. Acute stroke confers a significant increase in adverse cardiac outcomes during short- and long-term follow-up [1]. A subset of patients with stroke might be at higher risk of long-term adverse cardiovascular outcomes. Identifying these patients, ideally with a simple test or biomarker, can help to reduce their long-term risk of adverse events.

Troponin is a highly sensitive and specific marker for myocardial necrosis that is used in the diagnosis and prognosis of patients with acute coronary syndrome. However, troponin elevation has been documented in multiple clinical settings in the absence of ACS [2, 3]. Increase in troponin has been documented in all types of stroke including ischemic stroke and subarachnoid hemorrhage [4]. In a meta-analysis of 15 studies involving 2,901 patients, elevated troponins were documented in 18.1% patients with stroke that included patients with EKG changes suggestive of myocardial ischemia [3]. Troponin elevation has also been documented in acute stroke without any evidence of acute coronary syndrome [5].

While some studies have not shown any influence of positive troponins on adverse outcomes [6, 7], others have shown an increase in all-cause mortality with troponin elevation in acute stroke during in-hospital stay and short-term follow-up [8–14]. An increase in all-cause mortality has been documented in patients with and without ischemic EKG changes [8–11]. Di Angelantonio et al. [11] reported an increased incidence of composite end point of in-hospital death and nonfatal myocardial infarction in patients with acute stroke and positive troponins, compared to those with normal troponins. However, this study neither excluded patients with ischemic EKG changes on presentation, nor did it address the incidence of nonfatal myocardial infarction during follow-up. There is a paucity of data on long-term prognostic value of positive cardiac biomarkers in patients with stroke without acute coronary syndrome. Our study focuses on the impact of elevated troponins in non-ACS acute stroke setting on long-term cardiovascular outcomes.

## 2. Methods

We performed a retrospective review of 566 charts for patients admitted to Temple University Hospital (TUH) with a diagnosis of “acute cerebrovascular accident (CVA)” or “acute stroke” between 2008 and 2010. Approval for this chart review was obtained from the Institutional Review Board. Inclusion criteria required patients to have levels of cardiac troponin I (cTnI) measured within 24 hours of admission and an echocardiogram performed during that admission. 212 patients met inclusion criteria.

**Figure 1:**
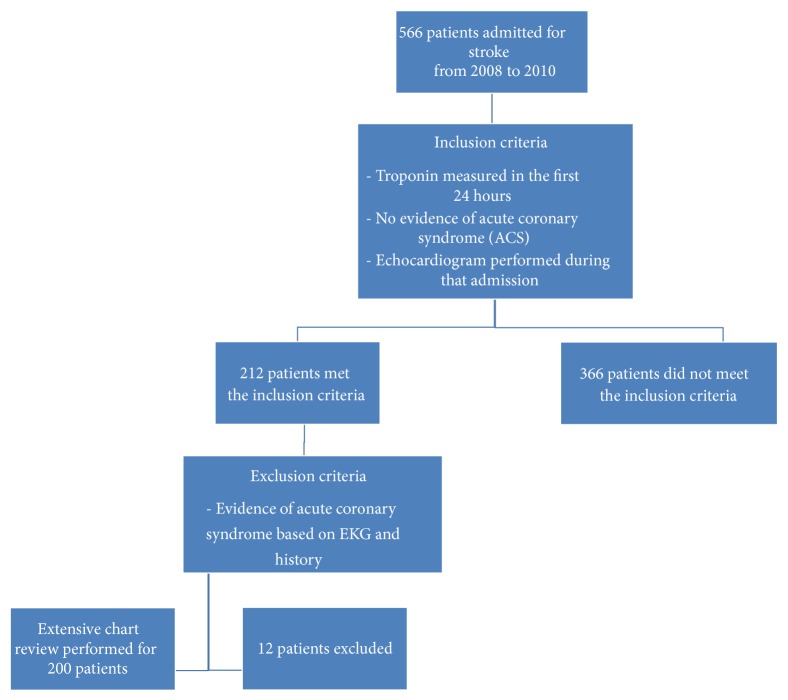
Distribution of patients selected for analysis in this study.

For patients that met inclusion criteria, if levels of CKMB were also measured, serum values for both biomarker subtypes were recorded. Patients were considered to have abnormal elevations in cardiac biomarkers if either CKMB (0.00–7.50 ng/mL) or cTnI (0.05–0.40 ng/mL) was above the hospital's normal reference level. Patients were excluded from the study if they had evidence of acute coronary syndrome in accord with the AHA/ACC guidelines [15]. ECGs were analyzed retrospectively by two separate physicians blinded to clinical outcomes. An ECG was determined to be consistent with ACS if there was ≥1 mm ST depression or ≥2 mm ischemic T wave inversion, new Q-waves, new left bundle branch block, or ST elevations consistent with ischemia. Of the 212 patients who met inclusion criteria, 12 were excluded because of suspected or confirmed ACS or death during admission (Figure 1). For the remaining 200 patients, prior medical history and key hospital data were recorded.

Using the electronic medical record, alpha imaging systems, centricity, PACS image viewer, and EPIC, both inpatient and outpatient medical records were reviewed to record baseline patient characteristics, length of hospital stay, and length of follow-up and to assess the time interval in days to the occurrence of a major adverse cardiac event (MACE). MACE was defined as nonfatal myocardial infarction, percutaneous coronary intervention (PCI), coronary artery bypass grafting (CABG), or death. Only the first admission was included for patients admitted more than once for a stroke. Chronic renal insufficiency was defined as baseline creatinine above 1.4 mg/dL.

### 2.1. Statistical Analysis

Categorical variables were analyzed using either the chi-square statistic or Fischer exact test, as appropriate. Continuous variables were expressed as mean and statistical significance was tested using Student's* t*-test. Patients were grouped as normal versus elevated based upon serum admission cTnI levels. Multivariate logistic regression models were constructed to determine variables that predicted abnormal elevations in admission serum cTnI. Similar models were constructed to identify variables that independently predicted long-term outcomes. Linear regression models were constructed to identify variables that predicted length of hospital stay, rehospitalizations, and restroke. Kaplan-Meir plots were used to assess effects over time. Associations were considered significant if alpha < 0.05. All analyses were performed with SPSS v21.0.

### 2.2. Measurement of Cardiac Troponins

The blood specimens analyzed in this study were collected in observation with routine precautions for venipuncture. Blood samples were allowed to clot completely prior to centrifugation and stored at room temperature (15 to 30°C) for no longer than two hours. Cardiac troponin I was measured by the Access AccuTni chemiluminescent immunoassay (Beckman Coulter). This assay uses two monoclonal antibodies in conjunction with alkaline phosphatase to bind antigenic sites in the solid phase, resulting in a complex between human cTnI and monoclonal anti-cTnI antibody. The Access AccuTni chemiluminescent immunoassay has a cTnI cutoff of 0.40 ng/mL; this cutoff yields the most optimal sensitivity and specificity.

### 2.3. Measurement of CK-MB

Similar to cTnI, CK-MB was also measured with the Access AccuTni chemiluminescent immunoassay, with a similar two-monoclonal antibody system.

## 3. Results

Demographic characteristics of the patient population based upon serum admission cTnI and their clinical measures at admission are shown in Table 1. Of 200 patients, 17 patients were found to have elevated cTnI. There were no statistically significant differences in age, sex, or ethnic makeup identified between the normal and elevated troponin groups. Patients with elevated admission serum cTnI had a greater prevalence of prior coronary artery disease when compared to their counterparts with normal levels (Table 1). During postdischarge follow-up (mean follow-up of 20.1 ± 10.3 months), patients with elevated serum cTnI had a significantly higher incidence of nonfatal myocardial infarction, MACE, and death from any cause when compared to patients with normal cTnI (Table 2).

### 3.1. Factors That Predict MACE

Table 2 shows the statistically significant increased incidence of adverse outcomes (nonfatal myocardial infarction, MACE, and death from any cause) in the elevated cTnI group.

Table 3 shows the contribution of clinical factors that predict MACE during the follow-up period. Using multivariate logistic regression, a statistically significant relation was only found for an elevated admission serum cTnI. Elevated CK-MB, prior history of coronary artery disease (CAD), diabetes (DM), hypertension (HTN), and chronic kidney disease (CKD) failed to show significant relationships with MACE. Also, newly depressed left ventricular ejection fraction (LVEF) found on echocardiogram did not predict MACE.

**Figure 2:**
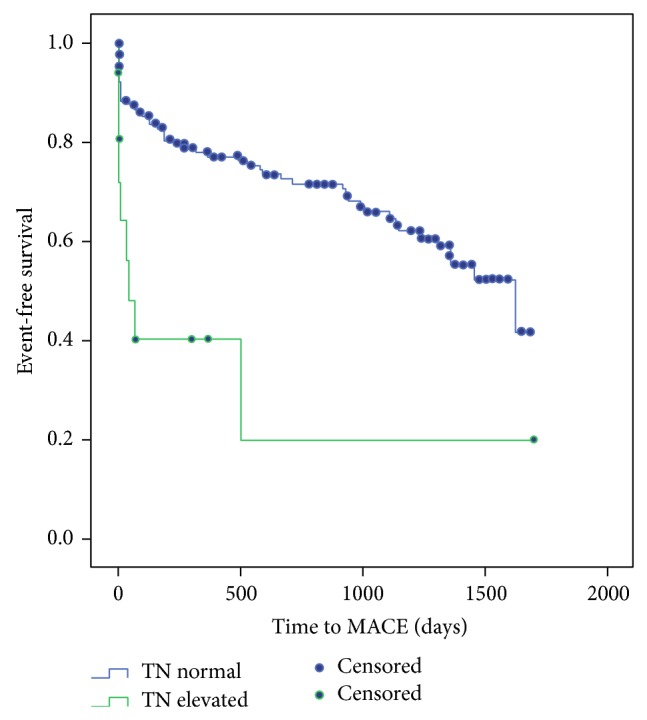
Kaplan Meier curves showing fraction of subjects with event-free survival over the study period (curves are significant with *P* < 0.001).

Figure 2 shows a significant difference in event-free survival from MACE when comparing patients with and without elevated admission serum cTnI.

### 3.2. Factors Predicting Length of Stay and Rehospitalizations

Table 4 shows thatelevated cardiac biomarkers (cTnI and CK-MB) do not predict length of hospital stay. Large stroke size is the only factor that approached significance for predicting the length of stay (*P* 0.09). Multivariate analysis also revealed that cardiac biomarkers (cTnI and CK-MB) or new cardiomyopathy (LVEF < 50%) does not predict rehospitalizations. Small stroke size is the only factor that approached significance for predicting number of rehospitalizations (*P* = 0.07).

### 3.3. Factors Predicting Restroke

Age, CAD, prior CVA, smoking, and atrial fibrillation did not predict incidence of restroke after discharge while hypertension showed a trend toward significance (*P* = 0.082).

## 4. Discussion

### 4.1. Mechanism of Troponin Elevation in Stroke

Masuda et al. [16] revealed in an animal model (18 dogs) of subarachnoid hemorrhage that, within five minutes of inducing a subdural hemorrhage, there was an elevation of noradrenaline and adrenaline, followed by elevation of troponin T and CK-MB, associated with decrease in cardiac output and new left ventricular wall motion abnormalities. Several studies have confirmed the presence of troponin elevation in high catecholamine states such as in ischemic stroke [1, 8–10, 14] and in takotsubo cardiomyopathy [17, 18]. The high concentration of catecholamines in the myocardium would bring about a calcium overload of myocardial cells [19] which can cause a reduction of myocardial contractility and can lead to an impairment of cardiac function due to the perfusion disturbance at the level of capillaries caused by an enhanced platelet aggregation [20].

### 4.2. Troponin Elevation Predicts Outcomes

Prognostic value of elevated troponin in acute CVA remains a controversial topic. Increased mortality has been predicted by elevated troponin in multiple studies [8–11]. However, it occurs in older sicker patients with similar risk factors for coronary disease and cerebral vascular disease. In most situations, it remains unclear if a patient had an acute myocardial infarction that leads to the stroke or the other way around. Thus, it is of paramount significance to identify if an elevated troponin in acute stroke setting is not associated with an acute coronary syndrome. In the absence of evidence for an ACS in an acute stroke, one should consider the long-term significance of an elevated troponin.

Multiple studies have documented that troponin elevation in the setting of acute CVA predicts an increase in all-cause mortality in different patient groups [8–11]. However, there is a paucity of data for the long-term significance of elevated cardiac biomarkers in a non-ACS setting in acute stroke. Jensen et al. [9] showed that elevation of cardiac troponin T in acute stroke in a non-ACS setting identifies a group of patients who are at increased risk of death from any cause over the following two years. However, it remains unclear if the increased risk is due to cardiac causes (myocardial infarction or heart failure).

Our study is unique because we focused on patients with an acute stroke but no evidence of acute coronary syndrome. In our study population, 20.5% had a prior history of CAD but, on multivariate analysis, elevated troponin was the only independent factor that predicted long-term cardiac outcomes over a follow-up period. We demonstrated that an elevated troponin in this setting identifies a high-risk patient group with increased risk of MACE (primarily due to nonfatal myocardial infarction) after discharge. This high-risk group of patients can benefit from aggressive monitoring and outpatient cardiovascular care to prevent worse outcomes. The other unique feature of our study is that, demographically, 63.5% patients in our study are African American.

### 4.3. New LV Dysfunction Does Not Predict MACE

The influence of systolic dysfunction has been proven to predict mortality in ischemic [21] and nonischemic cardiomyopathy [22]. However, in our study, newly diagnosed cardiomyopathy with left ventricular ejection fraction (LVEF) of less than 50% did not predict adverse outcomes. The likely cause is the influence of sympathetic surge during acute stroke that leads to transient depression of myocardial function rather than a persistently depressed LVEF. Thus, the pathophysiology of depressed LVEF in this setting may be very different from chronic heart failure involving the sympathetic nervous system and renin-angiotensin-aldosterone system [23].

### 4.4. Stroke Size Predicts Length of Stay and Rehospitalization

The only independent factor that predicted increased length of stay was large stroke size. It is intuitive that the larger the stroke size is, the worse the disability would be and the longer the recovery would be, leading to an increased hospital stay. However, elevated cardiac biomarkers did not predict longer hospital stay. On multivariate regression, we did not identify any specific factors that predict increased rehospitalizations except for a smaller stroke size. The likely explanation is that patients with small strokes were not very sick and lived long enough to have multiple rehospitalizations during the follow-up period.

### 4.5. Factors Predicting Re-CVA

The only factor we identified that predicted an increased risk of restroke was hypertension. Hyperlipidemia, diabetes, and CKD did not predict stroke recurrence. Well-known risk factors like atrial fibrillation and smoking did not predict risk of a restroke. Also, prior history of stroke and new systolic dysfunction did not predict restroke.

## 5. Study Limitations

This study was of single center and retrospective with a small sample size. There is an inherent bias that the troponins were checked in a sicker patient population. However, the impact was similar for both patient groups. Also, even with an extensive chart review to exclude patients with any evidence of acute coronary syndrome, the possibility of an active cardiac event during the hospitalization cannot be completely ruled out given the retrospective nature of the study. A prospective study with strict inclusion and exclusion criteria and a larger sample size is required to address this hypothesis in future.

## 6. Conclusion

Our study suggests that an elevated troponin level identifies a high-risk group among patients presenting with acute stroke and no evidence of acute coronary syndrome. This group of patients has higher long-term adverse outcomes independent of any prior history of coronary artery disease.

Larger prospective studies are needed to confirm these findings.

## Figures and Tables

**Table 1:** Descriptive statistics and demographic distribution.

Variable	Normal cTnI (*n* = 183)	Elevated cTnI (*n* = 17)	*P* value
Age	63.1 ± 10.8	62.7 ± 14	0.079
Male (%)	57.9	41.2	0.208
African Americans (%)	65.5	47.1	0.270
Smoker (%)	38.3	41.2	0.800
Hypertension (%)	79.2	64.7	0.217
Diabetes (%)	38.3	47.1	0.606
CAD history (%)	18.0	47.1	0.009
CKD (%)	5.5	17.6	0.086
Systolic BP (mmHg)	161.8 ± 23.2	153 ± 32.8	0.072
Hemoglobin (g/dL)	13.4 ± 2.6	12.2 ± 1.8	0.014
Creatinine (mg/dL)	1.24 ± 1.0	1.65 ± 1.6	0.143
Ldl (mg/dL)	119.9 ± 41	109.6 ± 34	0.249
Peak cTnI (ng/mL)	0.048 ± 0.06	2.04 ± 1.1	0.000
CK-MB (ng/mL)	3.23 ± 3.1	19.09 ± 13.8	0.000
LVEF (%)	50.2 ± 15.9	47.9 ± 21.6	0.008
Length of stay (days)	8.7 ± 13.2	15.3 ± 11.2	0.384

CAD: coronary artery disease. CKD: chronic kidney disease. LVEF: left ventricular ejection fraction. cTnI: cardiac troponin I.

**Table 2:** Incidence of adverse outcomes during postdischarge follow-up.

Variable	Normal cTnI (%)	Elevated cTnI (%)	*P* value
Nonfatal MI	3.3	41.2	0.0001
MACE	14.2	41.2	0.01
Death from any cause	14.5	41.2	0.017
Death from cardiovascular cause	0.5	5.9	0.163
CABG	1.1	0.0	0.837
Revascularization^*^	10.9	5.9	0.445
Rehospitalizations	53.6	35.3	0.205
Restroke	10.9	5.9	0.445

MI: myocardial infarction. MACE: major adverse cardiac events. CABG: coronary artery bypass graft.

^*^Revascularization during postdischarge follow-up due to new cardiac events.

**Table 3:** Multivariate logistic regression predicting MACE.

Variable	Odds ratio	95% CI low	95% CI high	*P* value
cTnI	9.760	2.419	39.378	0.001
CK-MB	0.28	0.055	1.44	0.128
HTN	2.294	0.741	7.106	0.15
DM	1.087	0.476	2.478	0.843
CAD	1.15	0.404	3.276	0.794
New LVEF <50%	1.164	0.460	2.942	0.749

HTN: hypertension. DM: diabetes mellitus. CAD: coronary artery disease. LVEF: left ventricular ejection fraction.

**Table 4:** Multivariable logistic regression predicting length of stay.

Variable	O.R.	95% CI low	95% CI high	*P* value
cTnI	2.360	−0.906	5.626	0.15
CK-MB	0.036	−0.16	0.232	0.718
New EF <50	−0.533	−4.529	3.463	0.790
Large stroke	2.535	−0.437	5.507	0.09

LVEF: left ventricular ejection fraction.

## Abbreviations

CVA: Cerebrovascular accident
ACS: Acute coronary syndrome
cTnI: Cardiac troponin I
CAD: Coronary artery disease
DM: Diabetes mellitus
MACE: Major adverse cardiac event
HTN: Hypertension
CK-MB: Creatine kinase-MB fraction.

## References

[1] Touzé E., Varenne O., Chatellier G., Peyrard S., Rothwell P. M., Mas J.-L. (2005). Risk of myocardial infarction and vascular death after transient ischemic attack and ischemic stroke: a systematic review and meta-analysis. *Stroke*.

[2] Agewall S., Giannitsis E., Jernberg T., Katus H. (2011). Troponin elevation in coronary vs. non-coronary disease. *European Heart Journal*.

[3] Agzew Y. (2009). Elevated serum cardiac troponin in non-acute coronary syndrome. *Clinical Cardiology*.

[4] Banki N., Kopelnik A., Dae M. W. (2005). Acute neurocardiogenic injury after subarachnoid hemorrhage. *Circulation*.

[5] Kerr G., Ray G., Wu O., Stott D. J., Langhorne P. (2009). Elevated troponin after stroke: a systematic review. *Cerebrovascular Diseases*.

[6] Whiteley W., Chong W. L., Sengupta A., Sandercock P. (2009). Blood markers for the prognosis of ischemic stroke: a systematic review. *Stroke*.

[7] Etgen T., Baum H., Sander K., Sander D. (2005). Cardiac troponins and N-terminal pro-brain natriuretic peptide in acute ischemic stroke do not relate to clinical prognosis. *Stroke*.

[8] Ay H., Koroshetz W. J., Benner T., Vangel M. G., Melinosky C., Arsava E. M., Ayata C., Zhu M., Schwamm L. H., Sorensen A. G. (2006). Neuroanatomic correlates of stroke-related myocardial injury. *Neurology*.

[9] Jensen J. K., Kristensen S. R., Bak S., Atar D., Høilund-Carlsen P. F., Mickley H. (2007). Frequency and significance of troponin T elevation in acute ischemic stroke. *The American Journal of Cardiology*.

[10] Jensen J. K., Atar D., Mickley H. (2007). Mechanism of troponin elevations in patients with acute ischemic stroke. *American Journal of Cardiology*.

[11] Di Angelantonio E., Fiorelli M., Toni D., Sacchetti M. L., Lorenzano S., Falcou A., Ciarla M. V., Suppa M., Bonanni L., Bertazzoni G., Aguglia F., Argentino C. (2005). Prognostic significance of admission levels of troponin I in patients with acute ischaemic stroke. *Journal of Neurology, Neurosurgery and Psychiatry*.

[12] Sandhu R., Aronow W. S., Rajdev A., Sukhija R., Amin H., D'aquila K., Sangha A. (2008). Relation of cardiac troponin I levels with in-hospital mortality in patients with ischemic stroke, intracerebral hemorrhage, and subarachnoid hemorrhage. *American Journal of Cardiology*.

[13] James P., Ellis C. J., Whitlock R. M. L., McNeil A. R., Henley J., Anderson N. E. (2000). Relation between troponin T concentration and mortality in patients presenting with an acute stroke: observational study. *British Medical Journal*.

[14] Faiz K. W., Thommessen B., Einvik G., Omland T., Rønning O. M. (2014). Prognostic value of high-sensitivity cardiac troponin T in acute ischemic stroke. *Journal of Stroke and Cerebrovascular Diseases*.

[15] Anderson J. L., Adams C. D., Antman E. M., Bridges C. R., Califf R. M., Casey D. E., Chavey W. E., Fesmire F. M., Hochman J. S., Levin T. N., Lincoff A. M., Peterson E. D., Theroux P., Wenger N. K., Wright R. S., Smith S. C., Jacobs A. K., Halperin J. L., Hunt S. A., Krumholz H. M., Kushner F. G., Lytle B. W., Nishimura R., Ornato J. P., Page R. L., Riegel B. (2007). ACC/AHA 2007 guidelines for the management of patients with unstable angina/non ST-elevation myocardial infarction: a report of the American College of Cardiology/American Heart Association Task Force on Practice Guidelines (Writing Committee to Revise the 2002 Guidelines for the management of Patients With Unstable Angina/Non ST-Elevation Myocardial Infarction): developed in collaboration with. *Circulation*.

[16] Masuda T., Sato K., Yamamoto S.-I., Matsuyama N., Shimohama T., Matsunaga A., Obuchi S., Shiba Y., Shimizu S., Izumi T. (2002). Sympathetic nervous activity and myocardial damage immediately after subarachnoid hemorrhage in a unique animal model. *Stroke*.

[17] Yoshimura S., Toyoda K., Ohara T. (2008). Takotsubo cardiomyopathy in acute ischemic stroke. *Annals of Neurology*.

[18] Scheitz J. F., Mochmann H. C., Witzenbichler B., Fiebach J. B., Audebert H. J., Nolte C. H. (2012). Takotsubo cardiomyopathy following ischemic stroke: a cause of troponin elevation. *Journal of Neurology*.

[19] Mann D. L., Kent R. L., Parsons B., Cooper G. (1992). Adrenergic effects on the biology of the adult mammalian cardiocyte. *Circulation*.

[20] Shattil S. J., Budzynski A., Scrutton M. C. (1989). Epinephrine induces platelet fibrinogen receptor expression, fibrinogen binding, and aggregation in whole blood in the absence of other excitatory agonists. *Blood*.

[21] Emond M., Mock M. B., Davis K. B. (1994). Long-term survival of medically treated patients in the Coronary Artery Surgery Study (CASS) registry. *Circulation*.

[22] Dec G. W., Fuster V. (1994). Idiopathic dilated cardiomyopathy. *The New England Journal of Medicine*.

[23] Francis G. S., Goldsmith S. R., Levine T. B., Olivari M. T., Cohn J. N. (1984). The neurohumoral axis in congestive heart failure. *Annals of Internal Medicine*.

